# Inorganic Nutrients Increase Humification Efficiency and C-Sequestration in an Annually Cropped Soil

**DOI:** 10.1371/journal.pone.0153698

**Published:** 2016-05-04

**Authors:** Clive A. Kirkby, Alan E. Richardson, Len J. Wade, Mark Conyers, John A. Kirkegaard

**Affiliations:** 1 Commonwealth Scientific and Industrial Research Organisation (CSIRO) Agriculture, GPO Box 1600, Canberra, Australia; 2 Charles Sturt University, E H Graham Centre for Agricultural Innovation, Wagga Wagga, New South Wales, Australia; 3 New South Wales Department of Primary Industries (NSW DPI), PMB Pine Gully Road, Wagga Wagga, New South Wales, Australia; Institution and Department: Université de Sherbrooke, CANADA

## Abstract

Removing carbon dioxide (CO_2_) from the atmosphere and storing the carbon (C) in resistant soil organic matter (SOM) is a global priority to restore soil fertility and help mitigate climate change. Although it is widely assumed that retaining rather than removing or burning crop residues will increase SOM levels, many studies have failed to demonstrate this. We hypothesised that the microbial nature of resistant SOM provides a predictable nutrient stoichiometry (C:nitrogen, C:phosphorus and C:sulphur–C:N:P:S) to target using supplementary nutrients when incorporating C-rich crop residues into soil. An improvement in the humification efficiency of the soil microbiome as a whole, and thereby C-sequestration, was predicted. In a field study over 5 years, soil organic-C (SOC) stocks to 1.6 m soil depth were increased by 5.5 t C ha^-1^ where supplementary nutrients were applied with incorporated crop residues, but were reduced by 3.2 t C ha^-1^ without nutrient addition, with 2.9 t C ha^-1^ being lost from the 0–10 cm layer. A net difference of 8.7 t C ha^-1^ was thus achieved in a cropping soil over a 5 year period, despite the same level of C addition. Despite shallow incorporation (0.15 m), more than 50% of the SOC increase occurred below 0.3 m, and as predicted by the stoichiometry, increases in resistant SOC were accompanied by increases in soil NPS at all depths. Interestingly the C:N, C:P and C:S ratios decreased significantly with depth possibly as a consequence of differences in fungi to bacteria ratio. Our results demonstrate that irrespective of the C-input, it is essential to balance the nutrient stoichiometry of added C to better match that of resistant SOM to increase SOC sequestration. This has implications for global practices and policies aimed at increasing SOC sequestration and specifically highlight the need to consider the hidden cost and availability of associated nutrients in building soil-C.

## Introduction

While soils are the largest sink for terrestrial C [1–3] many agro-ecosystems have lost approximately half (up to 50 t ha^-1^) of their original SOC over the last two centuries [4]. It is generally accepted that this loss has been a major factor leading to soil degradation and declining soil quality [5–7]. Restoring this “lost” SOC is a high priority for agricultural policy makers and practitioners across the globe. The Intergovernmental Panel on Climate Change identified agriculture as having one of the most significant near-term (by 2030) greenhouse gas mitigation potentials, with 90% of the mitigation potential arising from increased soil C sequestration [8,9]. They further stated that increased soil C sequestration would be a win-win situation as soil quality would also be improved. Moreover, enhancing sequestration deeper in the soil profile, where the ^14^C age suggests the C pool is older and more resistant to loss e.g. [10,11], may further increase the greenhouse gas mitigation potential of C sequestration.

In agricultural systems, above-ground crop residues, roots and root exudates are the primary source materials for SOM formation. However SOM is generally considered to be the organic fraction of soil, exclusive of coarse-fraction organic material that includes un-decayed plant and animal residues [12]. In this paper we define SOM as the fine-fraction component of SOM (FF-SOM <0.4 mm, as estimated by measuring FF-C) and is synonymous with heavy-fraction material (>1.4 g cm^-3^) that is commonly considered to be associated with a more stable and slowly decomposing pool of SOM [13,14]. We previously reported that FF-SOM in a wide range of surface soils globally has a near constant C:N:P:S ratio and, as a result of microbial processing, is more nutrient (NPS) rich than the fresh plant residues (e.g., wheat straw) from which it is derived. Using controlled laboratory conditions, we previously reported increases in the net humification of wheat straw added to soil (i.e. the net conversion of residue-C to FF-SOM-C) of two- to eight-fold (from 4.5–7.5% to 12.2–42.6%) across a range of soils (with varying clay contents and starting FF-C values) when inorganic-N, -P and -S were added with the straw. In addition, adding the inorganic nutrients with the straw resulted in an increase in the microbial biomass (mean 118%, range 39 to 177%) and an increase in the mass of straw decomposed (mean 85%, range 56 to 137%) during the incubation period. Rates of nutrient addition were designed to account for differences between the stoichiometric nutrient ratios found in wheat straw and that in the FF-SOM fraction [15].

In the previous soil incubation studies reported by Kirkby et al. [14,15] soil mesocosms were incubated under ‘ideal’ conditions of constant moisture, temperature and with thorough and regular mixing to ensure good aeration and intimate contact between soil and straw. In contrast, environmental factors vary in the field and may significantly affect the potential for C sequestration. First, the plant residues are typically larger when returned to the soil and the degree of mixing of the soil and crop residues would be expected to be less uniform in the field than that achieved in the laboratory. Second, soil temperature and moisture regimes also change diurnally and seasonally. Finally, under field conditions, inorganic nutrient additions may be subject to leaching or subsequent uptake by growing plants and would also be expected to be more unevenly distributed within the soil profile.

Increasing the FF-SOM in soil by enhancing the humification efficiency of crop-residues returned to soil under field conditions has wide implications for agricultural systems. More rapid breakdown of the residue by microorganisms and formation of new FF-SOM will inevitably require greater immobilisation of inorganic nutrients. Though the required amount of nutrients are relatively small, losses by leaching may also be minimised if the additional nutrients are effectively sequestered within the FF-SOM. Importantly, the stoichiometric ratios for FF-SOM used in our previous studies were largely based on surface soils, and it is also possible that these may change with soil depth. Previous studies have suggested a decline in C:nutrient ratios with depth as a result of soil properties and compositional change in microbial communities [16]. While fungi are generally the dominant microorganisms in surface soils bacteria, which are more nutrient rich than fungi per unit C, tend to dominate fungi in deeper soil layers which would result in a declining C:nutrient ratio with depth [17–19]. Subsequently this may affect the amount of nutrients required to sequester FF-SOM in the deeper layers.

In the present study we report on a 5-year field study (2007–2012) to test our central hypothesis that adding inorganic nutrients (NPS) to crop residues retained in the field, according to the stoichiometric requirements of FF-SOM, will increase humification efficiency and the C-sequestration potential. Identical amounts of C-rich post-harvest crop residues were incorporated each year into the soil with and without the addition of inorganic-N, -P and -S. Changes in the FF-SOM pool and total nutrient content of the soil were measured over time to a depth of 1.6m as were the C:N, C:P and C:S ratios. The specific hypotheses tested were (i) incorporating crop residues with nutrients will increase the size of the FF-SOM pool and total nutrient content of the soil compared to the control treatments, (ii) the C to nutrient (NPS) ratios will decline with depth consistent with changes in microbial composition (iii) nutrient addition to the incorporated residues will increase residue breakdown rate and (iv) adding inorganic nutrients to incorporated residues to increase C-sequestration will not lead to substantially increased nutrient (NPS) leaching.

## Materials and Methods

### Site details

The study was carried out on a private farm ("Oxton Park", Harden, NSW, Australia - 34^o^ 30’S, 148^o^ 17’E) a mixed farming enterprise owned by the O'Connor family for three generations. The authors gratefully acknowledge the O’Conner family for provision of the land during the trial. The site is on a well-drained soil near Harden in the south-eastern wheatbelt of Australia. The site was elevated (497 m a.s.l.) and sloping (3%) and the Red Chromosol soil [20] had a sandy-loam surface texture (clay 15%, silt 10% sand 75%) and pH (CaCl_2_) of 5.3. The site was part of a long-term field experiment established in 1990 to assess the effects of different tillage and stubble management treatments on soil fertility and crop performance in a continuous cropping system, as first reported by Kirkegaard et al. [21] and more recently by Kirkegaard et al. [22]. Crop management treatments (seven), including various tillage and crop residue treatments were replicated four times in a randomized block design. Individual treatment plots (30 m x 6 m) comprised two paired sub-plots (30 m x 2 m), side by side, separated by a central 1 m buffer to allow controlled-traffic management (no wheel traffic on plots). The experiment reported here only utilised one treatment—the residue incorporation treatment—in which crop residues were incorporated into the soil with an offset disc harrow to a depth of 0.15 m after the first rain following the annual summer harvest. From 2007 to 2012, one of each of the paired sub-plots (30 m x 2 m) in this treatment received supplementary nutrient addition each year at the time of residue incorporation, while the other sub-plot received no supplementary nutrient addition. The plots were cropped annually (May to December) with either wheat (*Triticum aestivum*) or in 2010 with canola (*Brassica napus*) for the 5 years of the experimental period between 2007 and 2012.

### Seasonal conditions

**Fig 1:**
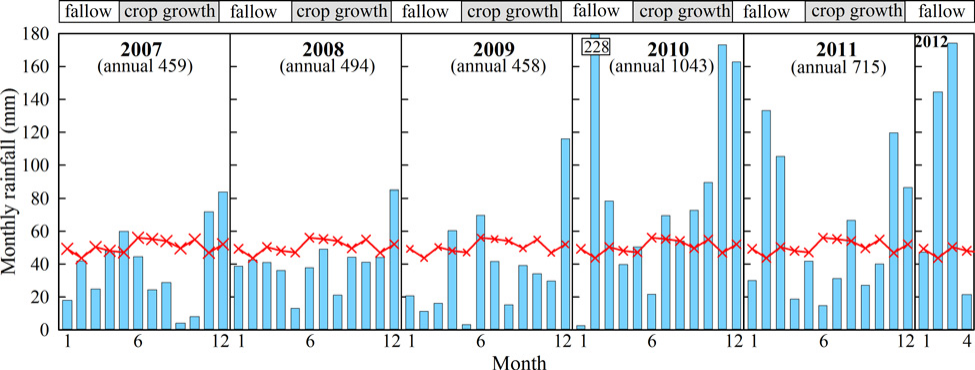
Measured monthly (blue shaded bars) and long-term mean (red crossed line) rainfall (mm) during the fallow and crop growth periods at the Harden field site.

The period of the experiment between 2006 and 2012 was characterised by below-average growing season rainfall (mid-May to November) which exceeded the long-term mean (364 mm) in 2010 only (Fig 1). In contrast, the rainfall after residue incorporation (wheat straw in all years, but canola in 2011) until time of sowing (approximately February to mid-May) was generally near to or above average which together with the warm summer temperatures (mean minimum 10°C, mean maximum 28°C) provided good conditions for the breakdown of incorporated residues.

Where applied, the supplementary nutrient additions generally increased the early vegetative biomass of the crops in the (+) nutrient treatments, perhaps suggesting some of the nutrients remained in plant-available forms and stimulated early growth. However, dry spring conditions allowed the slower-growing (−) nutrient treatments to generate similar biomass by anthesis, presumably as the (+) nutrient treatments had exhausted soil water and growth became limited by water availability. Low spring rainfall (September and October) during flowering and grain development also tended to reduce the yield potential of crops in most years. By final harvest, the levels of biomass and yield from the (+) nutrient treatment were significantly higher than the (−) nutrient treatment in only one of the five years of measurement. As a result of this water limitation to growth and yield (not uncommon in rain-fed dry-land environments), the outcomes of treatments on soil-C are not confounded by large differences in primary production, returned biomass or nutrients removed in harvested yield.

### Treatments

Prior to 2007, crop residues were routinely incorporated to a depth of 0.15 m with one or two passes of an offset disc harrow in late summer or early autumn when seasonal rainfall facilitated penetration of the discs. From 2007 to 2012 the incorporation of the residues was more thorough, using a flail mulcher to pulverise the standing residue and then a rotary cultivator to thoroughly mix and incorporate the material. The amounts of residue were carefully balanced across all plots after harvest, and prior to incorporation, to ensure uniform amounts were returned to the soil. This balance ensured that despite possible differences in crop residue production on the “plus nutrient” sub-plots (which occurred in 2 of 4 years), the same quantity of residue was incorporated into both sides of each replicate each year. If necessary, additional residue was sourced from buffers adjacent to the experimental plots. As soon as practicable after harvest (usually late January), the residue was balanced between sub-plots and mulched to produce pieces no more than 0.15 m long using the mechanical plot flail mulcher. Supplementary nutrients where then broadcast onto the surface of the mulched residue for the “plus nutrient” sub-plot. Following the first significant rains after residue mulching and supplementary nutrient addition, (approximately 10 mm or more in one day to soften the soil, which usually occurred in mid-February) the residue was incorporated to a depth of 0.10–0.15 m using a rotary cultivator to ensure that the soil and residue were thoroughly mixed with little residue apparent at the soil surface.

Each year the amount of inorganic nutrients (NPS) added to the residue was calculated as that required to humify 30% of the above-ground stubble-C according to the stoichiometric N requirements of the FF-SOM; C:N:P:S = 10,000:833:200:143 [14]. The residue nutrient analyses from the four replicates varied slightly each year but to ensure there were sufficient nutrients the nutrient analysis of the most nutrient poor residue was used for the calculation and the same amount of nutrients were added to each of the four replicates (Full details S1and S2 Tables). A commercially-available granulated fertiliser (Granulock 15, Incitec Pivot Fertilisers; N, P, S = 14.3, 12.0 10.5% respectively) was used to supply the nutrients. The granular fertiliser was pulverised (Labtechnics pulverising mill, model LM1, Adelaide, Australia) to produce a powder to enable a more even spread over the mulched residue. The amount of fertiliser added per unit of residue varied slightly each year depending on the N, P and S levels measured in the residue but was added at an approximate rate of 22 kg per tonne of stubble. As nitrogen is the element needed in the greatest amount for the humification process (833 units per 10,000 units of stubble-C), the amount of fertiliser added was calculated according to stoichiometric N requirements. However relative to the stoichiometric ratio for N, the commercial fertiliser contained an excess of P and S relative to the amount required for humification purposes. The cropping sequence, mean yields and residue loads produced are shown in Table 1, while the nutrient required as per the stoichiometric calculations and the actual nutrients applied each year of the experiment are shown in Table 2.

**Table 1:** Cropping year, crop (wheat, canola), quad yield (t ha^-1^), stubble loads (t ha^-1^), header yield (grain removed from paddock (t ha^-1^) for (+) and (−) nutrient treatments at harvest at the Harden field site (data are means and standard errors of the mean, SEM, *n = 4*).

		(−) nutrients			(+) nutrients		
Year	Crop	Quad yield	Stubble load	Header yield	Quad yield	stubble load	Header yield
2007^a^	W	2.9 *(0*.*16)*	7.0 *(0*.*47)*	2.4 *(0*.*13)*	-	-	-
2008	W	3.8 *(0*.*30)*	9.0 *(0*.*27)*	3.3 *(0*.*18)*	2.9 *(0*.*40)*	8.7 *(0*.*19)*	2.3 *(0*.*24)*
2009	W	3.2 *(0*.*17)*	8.0 *(0*.*40)*	2.8 *(0*.*09)*	3.1 *(0*.*21)*	9.4 *(0*.*77)*	2.4 *(0*.*11)*
2010^b^	C	2.7 *(0*.*27)*	5.6 *(0*.*78)*	-	4.3 *(0*.*27)*	10.6 *(0*.*23)*	-
2011	W	5.5 *(0*.*18)*	10.9 *(0*.*49)*	4.6 *(0*.*07)*	4.8 *(0*.*55)*	10.1 *(0*.*68)*	4.2 *(0*.*10)*

^a^2007 was the first year of the trial with stubble being produced so nutrient treatments could be applied the following season and as such there was no (+) nutrient treatment in 2007.

^b^No header harvest in 2010 due to mechanical issues; hand quadrats were taken for biomass and yield samples and the crop ploughed in.

**Table 2:** Calculated supplementary nutrients required (kg ha^-1^) each year to achieve 30% humification of above ground stubble-C on the (+) nutrient treatment at the Harden field site, and actual supplementary nutrients added (kg ha^-1^).

		calculated nutrients required			actual nutrients applied		
Year		N	P	S	N	P	S
2008		39	15	9	38	33	29
2009		50	16	8	50	42	36
2010		52	16	4	52	44	39
2011		65	21	-29*	65	54	48
2012		60	18	9	60	50	44
	Total	266	87	29	266	223	195

*2011 stubble was canola which had S in excess to that required to humify 30% of the stubble-C but this was not taken into account when calculating 2011 nutrients required for stubble-C humification.

### Sample collection, preparation and analyses

#### Soil cores and bulk density measurements

Soil cores (43 mm diameter) were taken from all treatments in 2006 to establish base-line measurements. Six cores, three from each sub-plot, extracted using a tractor-mounted hydraulic corer, were taken to a depth of 1.6 m from each replicate, and carefully separated into 10 cm increments which were bulked per plot for analysis and calculation of bulk density. In April 2012, a similar coring methodology was followed, however, the cores taken from the plus and minus nutrient sub-plots were kept separate for analysis.

Soil cores were also taken in 2010 for bulk density measurements from each of the four treatment blocks for the site. With the exception of the 0–10 and 10–20 cm layers, where the tillage treatment directly influenced the bulk density, the mean bulk density values of the four treatment blocks from cores taken on all three dates were used to estimate nutrient loads for each 10 cm layer from 20 to 160 cm (S3 Table). Due to the increase in cultivation intensity, and the possible change in near-surface bulk density, five new cores from each sub-plot were taken in 2012, in addition to the deep cores, to estimate bulk density for each of the four treatment blocks in the 0–10 and 10–20 cm layers for the experiment (S3 Table).

#### Soil preparation and analyses

The soils were prepared for analysis using a dry sieving/winnowing procedure described in detail in Kirkby et al. [13]. Briefly, the soils were air dried and gently crushed to separate soil, any plant material and gravel and were then passed through a 2 mm sieve to separate the soil from the gravel and any large pieces of plant material. The gravel was weighed and discarded, along with any plant material. Any coarse (> 0.4 mm) or light fraction-organic material that had passed through the 2 mm sieve was subsequently removed from the soil using the dry sieving/winnowing procedure described in detail in Kirkby et al. [13]. Organic matter remaining in the soil following this procedure was assumed to be heavy-fraction (>1.3 g cm^-3^) or fine-fraction SOM (FF-SOM, <0.4 mm) that is usually assumed to belong to the more stable, slowly-decomposing pool of SOM [13]. A 100 g sub-sample was subsequently pulverised (Labtechnics pulverising mill, model LM1, Adelaide, Australia) giving a grain size of approximately 50 μm to be used for later chemical analyses.

Total C and N concentrations were determined using a dry-combustion analyser (Europa Scientific Model 20–20, Crewe, UK). Total acid-extractable P and S concentrations were determined by inductively-coupled plasma optical emission spectroscopy (ICP-OES, Varian Vista-Pro (axial) Melbourne, Australia) following microwave-assisted acid digestion using reverse aqua-regia, 0.5 g soil, 9 ml concentrated nitric acid and 3 ml concentrated hydrochloric acid, according to method 3051A of the USEPA (1998). FF-C, -N, -P and -S loads were calculated from the measured FF-C, -N, -P and -S concentration values after adjusting for the measured gravel content and bulk density (S3 Table).

#### Plant sampling and nutrient analysis

Crops (wheat or canola) were sown each year of the experiment in autumn using local agronomic recommendations and varieties. At harvest the total crop biomass was measured using 2 m x 0.4 m bordered quadrat cuts of plant material removed at ground level and oven dried. The biomass was separated into grain and residue (Table 1). Immediately following the quadrat cuts the whole plot was harvested using a plot harvester. Some of the smaller grain was discarded out the back of the harvester, along with the crop residues, thus the grain removed from the paddock, the header yield, was always slightly less than the quadrat yield (Table 1). The nutrient content of the plant material was determined using the same methodology as described above for the soil (C and N, dry combustion analyser; P and S, ICP-OES following microwave-assisted acid digestion using reverse aqua-regia). The measurements of final plant biomass and residue and grain nutrient concentrations (Full details S1 Table) were used to develop nutrient budgets at the site and to allow mass balance for nutrients to be calculated across the 5 years of the experiment.

#### Nutrient (NPS) mass balance

Although there was no attempt to measure leaching losses, a calculation was made of the NPS mass balance to assess whether it was likely that there was any large NPS leaching losses, especially from the (+) nutrient treatment. Fertiliser additions (be it for normal crop requirements or supplementary nutrients added to increase the crop residue humification efficiency), NPS added as the extra straw to the (−) nutrient treatment and mean nutrients removed in grain from the two nutrient treatments were measured. These were balanced against the NPS loads measured in 2006 to estimate what the theoretical loads in 2012 should have been without any leaching losses. These loads were then compared with the actual loads measured in 2012 in both nutrient treatments and an assessment made as to whether large amounts of NPS were unaccounted for.

### Statistical analyses

The data for the distribution of C (and NPS) throughout the profile (e.g. the concentrations and total loadings from 0 to 160 cm) were not normally distributed and a non-parametric test (Wilcoxon signed rank test) was used to assess differences between 2006 and 2012. In contrast, the grouping of C (and NPS) within defined depth intervals (e.g. the concentrations in the 0–10 cm interval, or the loadings in the 10–30 cm interval) were normally distributed within each defined depth interval, and a paired t-test was used to assess if there were differences between 2006 and 2012 for each depth interval separately. A Pearson correlation analysis was used to compare the rates of change of the three ratios (C:N, C:P and C:S) with depth with the rationale that a strong correlation between the rates of change with depth for the different ratios, if any, could indicate a common mechanism for the changes. In contrast, a weak or no correlation, would indicate that any rate changes in the individual ratios with depth were not related.

## Results

### Residue breakdown rate

In 2008, the wheat residue was incorporated on February 8 and the crop sown on 15 May. Immediately prior to sowing, one soil sample from each of the four (+) and (−) nutrient treatment sub-plots (from an area of 0.1 m^2^ and to 0.15 m depth) was taken to assess whether nutrient addition altered the residue breakdown rate. The plant material remaining in the soil was removed using the dry sieving/winnowing procedure [13], brushed free of any soil, and weighed. On average only 24% (±1.9) of the initial residue load (8.9 t ha^-1^) remained in the (+) nutrient treatment plots while 88% (±3.9) remained in the (−) nutrient plots.

### Soil FF-C, -N, -P and -S concentration

**Fig 2:**
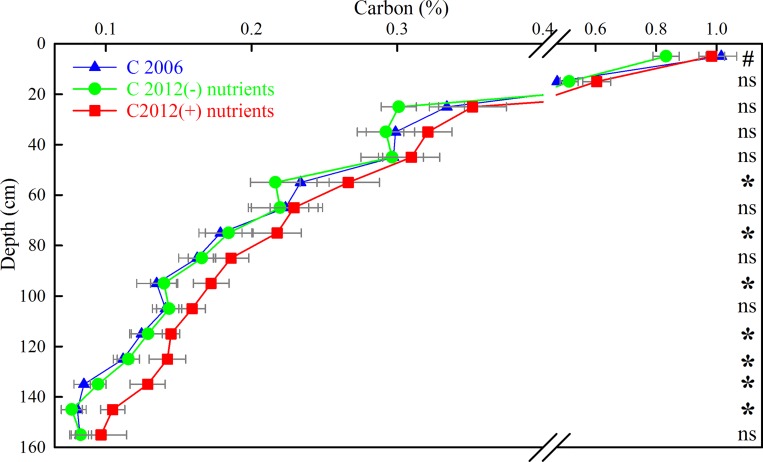
Mean FF-C concentration (%) to 1.6 m depth in the <2 mm soil fraction in 2006 and in the (+) and (−) nutrient treatments in 2012. Data are means and SEM, *n = 4*. (# and * indicate a significant difference between the 2006 value and the 2012 value for the (−) and (+) nutrient treatments, respectively P<0.05; ns = not significant)

There was a significant increase in the median FF-C concentration (P<0.001) in the (+) nutrient plots, when considering the 1.6 m soil profile in its entirety, when measured in 2012 compared with the initial levels in 2006, although the difference was not significant at all soil depths (Fig 2, full details S3 Table). Half of the depth increments that showed a significant increase in FF-C concentration were below 1 m. Over the entire 0–1.6 m soil profile there was no significant change in the FF-C concentration for the (−) nutrient plots in 2012 compared with 2006 (P = 0.485), although there was a significant decrease in the FF-C concentration in the 0–0.1m layer (P = 0.00156, Fig 2, full details S3 Table).

**Fig 3:**
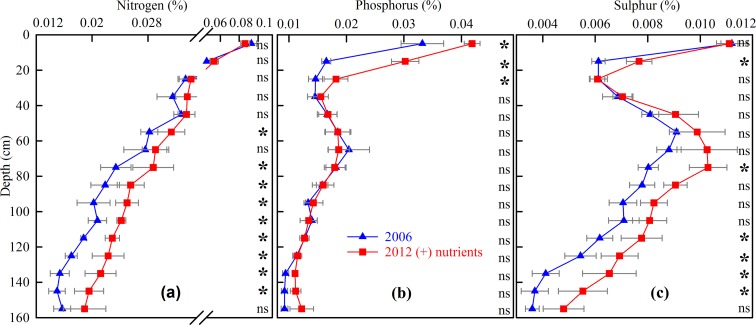
Mean FF-N, -P, -S concentrations (%) to 1.6 m depth in the <2 mm soil fraction in 2006 and the (+) nutrient treatment in 2012. Data are means and SEM, *n = 4*. (* indicate a significant difference between 2006 value and 2012 value from (+) nutrient treatment P<0.05; ns = not significant)

Similarly, there was a significant increase in FF-N, -P and -S concentrations (P<0.001 for all three) in the (+) nutrients plots, when considering the 1.6 m soil profile in its entirety, compared to 2006 levels, but again, this difference was not significant at all soil depths. A majority of the depths that showed a significant increase in FF-N or FF-S were in the deeper layers (Fig 3A and 3C respectively). In contrast to FF-N and -S, there was no significant difference in mean FF-P concentration in the deeper layers, compared with 2006 levels, but there was a significant increase in the FF-P concentration in the upper 0.3 m of the soil profile only (P≤0.022, Fig 3B). Consistent with the FF-C concentration across depths, there was no significant change in the FF-N, -P and -S concentrations in the (−) nutrient treatment over the 1.6 m soil profile between 2006 and 2012 (P>0.05, data not shown), but the 2012 levels of FF-N, -P and -S were lower in the 0–0.1m layer (P<0.01, P<0.05, P<0.05 respectively) (Full details S3 Table).

### Soil FF-C, -N, -P and -S stocks

When considering the 1.6 m soil profile in its entirety the mean stock of soil FF-C in the (+) nutrient treatment, increased by an average of 5.5 t ha^-1^ (P = 0.004, Table 3, full details S3 Table) in 2012 as compared to the level in 2006, with approximately 59% of the FF-C occurring below 0.3 m. Similarly the FF-N, -P and -S stocks in the (+) nutrient treatment increased by 695 (P = 0.005), 413 (P = 0.034), 277 (P<0.001) kg ha^-1^ respectively. In contrast, while there was little change in the FF-C stock of the (−) nutrient treatment at most depths between 2006 and 2012 (P>0.05 for CNP and S), there was a significant decrease of 2.9 (P<0.001) t ha^-1^ in FF-C in the 0–0.1 m layer and an associated decrease in FF-N, -P and -S stocks of 307 (P<0.001), 100 (P = 0.019) and 36 (P = 0.006) kg ha^-1^ respectively (Table 3, full details S3 Table).

**Table 3:** FF-C, -N, -P and -S stocks (t ha^-1^) at the Harden field site in 2006 and for the (−) and (+) nutrient treatments in 2012. Data are means and SEM. *n = 4*.

	C stock			N stock			P stock			S stock		
depth (cm)	2006	2012(−)	2012(+)	2006	2012(−)	2102(+)	2006	2012(−)	2012(+)	2006	2012(−)	2012(+)
0–10	13.2 *(0*.*9)*	10.3 *(0*.*9)*^b^	12.1 *(0*.*8)*^b^	1.21 *(0*.*10)*	0.90 *(0*.*09)*^b^	1.07 *(0*.*08)*^a^	0.43 *(0*.*05)*	0.33 *(0*.*03)*^a^	0.52 *(0*.*03)*^a^	0.15 *(0*.*01)*	0.11 *(0*.*01)*^a^	0.14 *(0*.*01)*
10–30	12.4 *(0*.*8)*	11.9 *(0*.*8)*	14.0 *(1*.*3)*	1.18 *(0*.*08)*	1.15 *(0*.*05)*	1.29 *(0*.*09)*	0.47 *(0*.*03)*	0.52 *(0*.*05)*	0.71 *(0*.*06)*^a^	0.19 *(0*.*01)*	0.18 *(0*.*01)*	0.20 *(0*.*01)*
30–60	12.3 *(0*.*6)*	11.9 *(1*.*0)*	13.3 *(0*.*8)*	1.37 *(0*.*06)*	1.36 *(0*.*12)*	1.46 *(0*.*08)*	0.74 *(0*.*08)*	0.74 *(0*.*09)*	0.76 (*0*.*08)*	0.36 *(0*.*01)*	0.36 *(0*.*02)*	0.39 *(0*.*03)*
60–90	8.7 *(0*.*9)*	8.8 *(1*.*1)*	9.8 *(1*.*0)*^a^	1.13 *(0*.*12)*	1.18 *(0*.*11)*	1.29 *(0*.*12)*^a^	0.85 *(0*.*12)*	0.79 *(0*.*09)*	0.82 *(0*.*10)*	0.38 *(0*.*01)*	0.37 *(0*.*03)*	0.46 *(0*.*03)*
90–120	6.2 *(0*.*3)*	6.4 *(0*.*2)*	7.4 *(0*.*5)*^a^	0.92 *(0*.*05)*	1.04 *(0*.*05)*	1.12 *(0*.*05)*^a^	0.62 *(0*.*04)*	0.58 *(0*.*04)*^a^	0.63 (*0*.*07)*	0.32 *(0*.*03)*	0.31 *(0*.*03)*	0.38 *(0*.*03)*
120–160	5.2 *(0*.*5)*	5.4 *(0*.*4)*	6.9 *(0*.*9)*^a^	0.91 *(0*.*07)*	1.02 *(0*.*12)*	1.19 *(0*.*17)*^a^	0.57 *(0*.*05)*	0.68 *(0*.*04)*	0.66 *(0*.*04)*	0.25 *(0*.*03*	0.27 *(0*.*03)*	0.35 *(0*.*07)*^a^
**0–160**	**58.0** *(1*.*7)*	**54.8** *(3*.*0)*	**63.5** *(3*.*9)*^a^	**6.72** *(0*.*16)*	**6.65** *(0*.*33)*	**7.42** *(0*.*34)*^a^	**3.68** *(0*.*28)*	**3.63** *(0*.*23)*	**4.09** *(0*.*28)*^a^	**1.63** *(0*.*05)*	**1.59** *(0*.*09)*	**1.91** *(0*.*06)*^a^

^a^ or ^b^ indicates a significant difference between the marked measurement and the relevant measurement in 2006

^a^ = P<0.05

^b^ = P<0.01.

### Nutrient (NPS) balance

The difference between the measured and calculated theoretic (without leaching) FF-N, -P and -S stocks in 2012 (Table 4) was approximately ±8%, which we consider to be reasonable for a field experiment of this type. More N was removed in grain from the (−) nutrient treatment than the combined total amount applied as inorganic fertiliser (for crop requirements) plus the nutrients in the extra residue added to the (−) nutrient treatment plots to even up the stubble loads. Although more fertiliser P and S was added to the (−) treatment than removed in grain, the measured FF-N, -P, -S stocks in the (−) nutrient treatment were lower than the calculated levels, and lower than the 2006 levels (Table 4).

**Table 4:** N, P, S balance (kg ha^-1^) for the (−) and (+) supplementary nutrient treatments in 2012 compared to the 2006 starting values.

	(−) nutrients			(+) nutrients		
	N	P	S	N	P	S
2006 values	6722	3675	1629	6722	3675	1629
normal crop fertiliser additions	234	75	39	234	75	39
supplementary NPS additions to aid humification	0	0	0	266	223	195
nutrients in extra straw added to (-) nutrient plots	46	5	29	0	0	0
nutrients removed in grain	-342	-41	-20	-331	-39	-19
theoretic 2012 levels	6660	3714	1677	6891	3934	1844
actual 2012 levels	6650	3631	1592	7417	4088	1906
difference between actual and theoretical 2012 levels	-10	-83	-85	526	154	62
% difference between actual and theoretical 2012 levels	-0.1	-2.2	-5.0	+7.6	+3.9	+ 3.3

In contrast, in the (+) nutrient treatment, more inorganic-N, -P and -S was applied than removed in grain (Table 4, full details S1 and S4 Tables) and the FF-N, -P and -S stocks, as well as the FF-C stock, increased compared to the 2006 levels. While the measured FF-N, and -P and -S, loads were all higher than the calculated theoretical amounts we consider the differences to be within acceptable limits for field experiments of this type.

### C:N:P:S ratios

**Fig 4:**
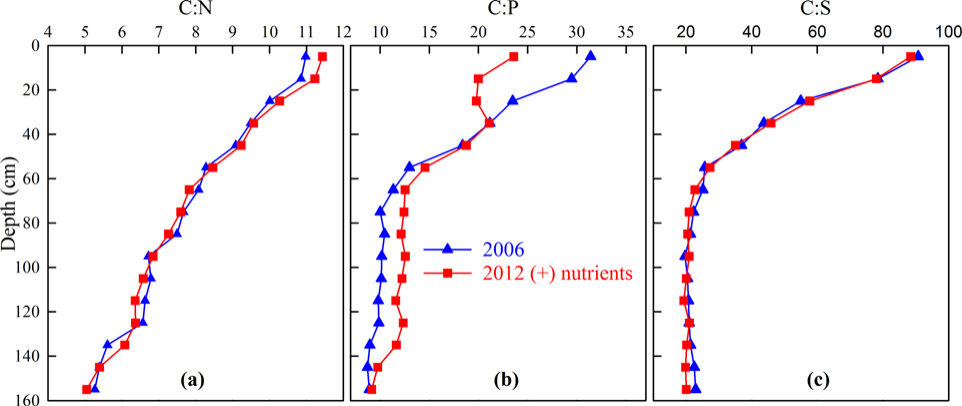
FF-C:N, -C:P, -C:S ratios to 1.6 m depth in the <2 mm soil fraction in 2006 and the (+) nutrient treatment in 2012. Data are means, *n = 4*.

The mean soil C:N, C:P and C:S ratios all declined with depth (Fig 4A, 4B and 4C respectively, full details S3 Table) when measured in 2006. In 2012, in the (−) nutrient treatment, the ratios similarly declined and the pattern of decline was comparable to 2006 (S3 Table). Despite a general increase in FF-C, -N and -S concentrations in the (+) nutrient treatment by 2012, the C:N and C:S ratios in 2012, at all depths to 1.6 m, were the same as the 2006 values (Fig 4A and 4C respectively). The C:P ratio in the (+) nutrient treatment was similar to the 2006 values at most depths but were lower near the surface (Fig 4B). There was a significant positive correlation between the declining C:N, C:P and C:S ratios with depth in 2006 and in both treatments in 2012 (Table 5).

**Table 5:** Correlation between the changing FF-C:N, -C:P and -C:S ratios with depth (0 to 1.6 m) in 2006 and for the (−) and (+) nutrient treatments in 2012, *n = 16*.

	2006		2012			
	nutrients		(-) nutrients		(+) nutrients	
	C:P	C:S	C:P	C:S	C:P	C:S
C:N	0.926	0.858	0.952	0.899	0.956	0.900
C:P		0.978		0.962		0.891

*P<0.001 in all cases.

## Discussion

### Nutrients increased C sequestration throughout the soil profile

Nutrient addition to incorporated residue over a five year period increased the FF-C pool to 1.6 m by 5.5 t ha^-1^ compared to a decrease of 2.9 t ha^-1^ from the 0–10 cm layer when supplementary nutrients were not applied, despite the same amounts of residue being incorporated in both treatments. This is consistent with previous results achieved in laboratory incubations [14,15]. As would be expected from the stoichiometric hypothesis underlying this work, there was also a concomitant increase in the concentration of FF-N, -P and -S. In the (+) nutrient treatment, more inorganic-N, -P and -S fertiliser was added than removed in grain and we consider this reaffirms that N, P and S, as well as an adequate supply of C, are needed to increase the size of the FF-SOM pool, and therefore the FF-C pool [23]. There is a growing body of evidence that FF-SOM is largely composed of microbial debris e.g. [24,25] and we have previously shown, albeit under controlled conditions, increased microbial biomass when crop residues where incorporated with additional nutrients compared to incorporation of the same amount and quality of residue without supplementary nutrient addition [15]. Thus, when nutrients are added with crop residues in the field it is likely that they may be captured by the microbial biomass and not leached from the system as might be expected in a relatively coarse soil in the absence of an actively growing crop. This supports the suggestion by Neff et al. [26] that the potential loss of mineral N from soil is controlled to a large extent by biological factors, and that as long as demand for N is high, such as in a soil/residue mix where the residue degrades at a fast rate and is associated with higher microbial biomass, losses will be minimal.

The depth of increase in soil-C extended well below that to which the residue was incorporated with large and significant increases in FF-C, -N and -S occurring to a depth of 1.6 m. These large increases in FF-C in the deeper layers are perhaps not as surprising as they may first appear, given several recent studies suggesting that deep soil-C is a significant contributor, if not the major contributor, to the total soil-C pool. For example, Rumpel and Kogel-Knaber [27] reported that more than half of the total soil-C stocks world-wide are in sub-soil horizons while Harper and Tibbett [28] found that total soil-C was 2–5 times greater when sampling to 5 m compared to sampling to 0.5 m. Similarly, Johnson et al. [29] found that, when sampling to 1.2 m, 36–51% of soil-C was below 0.2 m in non Spodosols, while 66% was below 0.2 m in Spodosols.

There would seem to be at least three explanations for the accumulation of FF-SOM at depth following the incorporation of crop residues near the surface. Namely; (i) the newly formed FF-SOM could have been formed near the surface and then moved down the profile in a dissolved or fine particulate form, (ii) it may derive directly from increased root growth and fine root turnover at depth, especially in (+) nutrient treatments, or result directly from rhizodeposition and subsequent microbial transformation, or (iii) it may derive *in situ* by microbial transformation at depth following downward translocation of soluble SOM and inorganic nutrients following enzymatic degradation of the coarse plant residue material near the surface.

The formation of new FF-SOM near the surface followed by translocation to depth is probably the least likely explanation for the accumulation of the new deep FF-SOM, as the C:N, C:P and C:S ratios in surface soils were markedly different to those at depth. The higher early vigour of crops in the (+) nutrient treatments may well have been accompanied by more vigorous root growth, though no direct measurement of roots were made during the experiment. If so, it is also possible that a greater root density at depth may have increased nutrient levels through root turnover and via rhizodeposition, processes that can represent a large proportion of assimilated C in wheat (>50%) during the vegetative stage [30].

We believe that in situ formation of FF-SOM formation deeper in the profile is a more likely explanation. The primary saprotrophs (fungi, bacteria and archaea) responsible for the initial breakdown of particulate plant material are osmotrophs. They do not ingest particulate organic matter directly and must obtain substrates for metabolism and growth through the uptake of soluble materials. They secrete specific extracellular enzymes (e.g. cellulases, proteases, ligninases, amylases etc.) that are able to solubilise the complex molecules and polymers (e.g. cellulose, proteins, lignin, starch etc.) that comprise plant residues. Following this hydrolytic degradation small molecules and substrates are produced that can then be readily utilised by the primary saprotrophs for metabolism and growth [31]. Dighton [32], suggests that all such systems are “leaky” and that this leakiness is a result of inefficiencies in utilising the soluble end products of this enzymatic degradation. He suggests that the primary saprotrophs don’t absorb all of the soluble end products they produce and some will be accessible to other organisms. In 2008 in the (+) nutrient treatment, 76% of the particulate crop residues had disappeared in the three month period between residue incorporation and sowing. Presumably this is due, in part, to the solubilising action of the exoenzymes released by the primary saprotrophs as described above. In addition, the soil at this site is relatively coarse and nutrients may be subject to leaching [33], which could have been further exacerbated by the increased intensity in cultivation [34]. Substantial rainfall during this period of residue breakdown is normal (Fig 1) and it is likely that some of the solubilised material originating from the degraded residues, along with some of the newly added inorganic nutrients may have leached deeper into the soil profile to provide nutrition for deeper microorganisms. There is evidence of substantial and active microbial communities at depth in some sites. For example, Vendkamp et al. [35] showed that although microbial activity was highest in the top 0.3 m, it contributed only 50% to the total microbial activity to a depth of 3 m. Leaching of solubilised material, prior to it being utilised by the saprotrophs higher in the profile responsible for its solubilisation, could therefore provide microbial communities at depth with energy and nutrients [34] eventually leading to the deep in-situ formation of FF-SOM. The use of labelled plant residues and the tracking of the labelled solubilised end products could be used to test this hypothesis. Irrespective of the mechanism, the increase in C sequestration by provision of supplementary nutrients and the apparent avoidance of gross leaching losses suggests the methodology employed here may be an effective mechanism to improve soil through FF-SOM increases throughout the soil profile. In addition this deeper translocation of C needs to be considered when estimating C loss to the atmosphere, especially following cultivation combined with residue retention, where shallow sampling for soil-C may underestimate the total soil-C stock, and over-estimate presumed C losses as CO_2_ emissions.

In contrast to the (+) nutrient treatment, and despite approximately 20 t ha^-1^ of above-ground residue-C being returned to the soil over five growing seasons (with potentially even more returned in root matter and root exudates) the FF-C stock in the top 0.1 m declined by 2.9 t ha^-1^ in the (−) nutrient treatment compared to 2006 levels with no significant change below 0.1 m. This loss of FF-C, despite the large amount of residue-C added indicates a possible priming effect as discussed by Kirkby et al. [15] and Fontaine et al. [36]. There was also a concomitant decline in the FF-N, -P and -S stocks in the 0.1 m layer which reaffirms the need for adequate N, P and S, along with a supply of new C, to form new FF-SOM, and/or to retain pre-existing FF-C. More fertiliser P and S was added than removed in grain, suggesting that any losses of P and S from this system may have been by leaching. In contrast, more N was removed in grain than applied as fertiliser reflecting modern farming systems, where the aim is to apply the minimum amount of fertiliser N to achieve the expected yield by relying on soil mineralisation to provide a portion of the required N budget.

### Implications for improved fertiliser strategies and the “nutrient-use-efficiency” paradigm

When assessing crop fertiliser N requirements, it is common to estimate total N requirements (based on crop type, soil type, seasonal conditions etc.), inorganic-N in the soil at sowing and N that may become available as previous crop residues decompose (i.e. mineralisation), and to apply the difference as ‘new’ fertiliser N. As more N was removed in grain in the (−) nutrient treatment, than the combined amount added as fertiliser and residue to this treatment, crops must have utilised N mineralised from pre-existing FF-SOM and/or from decomposing crop residues. By relying on N mineralised from existing organic matter in this way, the system was effectively being “mined” for N. This reliance explains why FF-C was lost from the (−) nutrient system despite the large additions of C-rich, but N-poor crop residues over the course of the experiment. Nevertheless, as the crops in the (+) nutrient treatment often showed increased early vigour, compared to the (−) nutrient treatment, (which was often not converted into increased yield due to low moisture availability during grain fill), there may have been an increased C input from larger root systems and greater root exudates. This does not remove the need for N, P and S in quite precise amounts (at least near the surface) to form FF-SOM. Even with large inputs of crop residue-C, without these additional nutrients the FF-SOM pool will not increase in size, as observed here, and may even decrease [15,36].

### Declining nutrient ratios with depth

Numerous studies have shown declining soil C:N ratios with depth e.g. [37–39] and as indicated by Rumpel and Kogel-Knaber [27] this may not be unexpected. Baisden et al. [40] suggested that a decrease in C:N ratio may be due to increased ammonia fixation in clay minerals which often increases with depth e.g. [38,39], possibly explaining the observed decreases in C:N ratio that occur with depth. However, the amount of ammonium fixation required to explain some of the significant C:N ratio decreases reported e.g. as observed here and [37–39] seem unrealistic.

While an increase in ammonia fixation in clay minerals may contribute to a decrease in the C:N ratio with depth [40], this cannot explain a decrease in the C:P or C:S ratios. The significant positive correlations we found between the rate of decline in the C:N, C:P and C:S ratios with depth suggests that there may be a common mechanism to explain the majority of the decreases. As discussed by McGill et al. [16] the declining C:nutrient ratios with depth may be due to differences in the composition of the microbial population with depth. Bacteria are more nutrient rich than fungi, with more N, P and S per unit of C. The C:N ratios of fungi and bacteria have been reported to be in the range 8–25 and 5–10 respectively [41,42]; the C:P ratio 300–1190 for fungi and 5–370 for bacteria [43] and the C:S ratio 54–192 for fungi and 31–94 for bacteria [44,45]. McGill et al. [16] reported that the C:N ratio in their system reached a maximum when fungi dominated, but declined as bacteria became dominant. Further research to estimate the relative contribution made by each of these groups to the FF-SOM composition at different depths, perhaps using amino sugar assays or molecular techniques, is needed to investigate this possibility. However, what is clear from the declining C:N, C:P and C:S ratios is that at this site more nutrients (NPS) are required per unit of FF-SOM found at depth, where it may be more resistant to loss, than FF-SOM sequestered near the surface e.g. [10,11]. We have previously reported that sequestering 1000 units of FF-C requires the sequestration of approximately 90, 19 and 14 units of N, P and S respectively [23]. The results from this field experiment largely confirm these estimates for N and S for near-surface soils with 90 and 12 units of N and S respectively required for each 1000 units of FF-C in the upper 0.2 m. To a depth of 1.6 m, however, the results from this study suggest that 195 units of N and 42 units of S are required for each 1000 units of FF-C at that depth. By contrast, we cannot make an estimate of the P required for each 1000 units of FF-C from this study, as we only analysed the FF-SOM for acid extractable-inorganic P rather than organic-P, which has been shown to be a more reliable parameter for estimating the amount of P associated with each unit of FF-C [13].

## Conclusions

Findings of this field study are consistent with the findings of previous laboratory incubations [14,15], that an adequate supply of N, P and S can significantly increase the proportion of crop residue-C sequestered as FF-SOM-C. Our approach did not target primary plant productivity or C input (which was held constant) as the mechanism to improve C sequestration, but rather the humification efficiency of the soil microbiome as a whole, by deliberately manipulating the C:N:P:S ratio of added inputs. This explains the conundrum of the surprisingly low C sequestration under many long-term crop residue retention systems where the balance of nutrients is not considered along with C input. It also calls into question policies related to soil-C sequestration that do not account for the supply and value of the stabilising inorganic nutrients required, particularly if the sequestration is expected to occur deeper in the soil profile. In addition, current “nutrient-use efficiency” approaches to crop nutrition may inadvertently limit the supply of nutrients for the formation of new FF-SOM, by applying only sufficient nutrient for crop uptake. Furthermore, a lack of available nutrient supply following the addition of high energy C-rich crop residue may indeed exacerbate the mineralisation of pre-existing FF-SOM, via a priming effect, causing an overall net loss of FF-SOM. However, it must also be recognised that the nutrients need to be supplied in balance, otherwise those that cannot be sequestered into the FF-SOM pool may be prone to loss.

Our methodology, based on the provision of supplementary inorganic nutrients to meet the stoichiometric ratio requirements of SOM to achieve a humification efficiency of 30%, was effective at increasing soil-C by up to 5.5 t C ha^-1^ over a five year period, and reversed a declining trend in soil-C that continued where supplementary nutrients were not applied. The mass balance suggests there was little if any nutrient loss from the system that could contribute to adverse environmental outcomes but further research is needed to quantify and validate this. The intensive cultivation used for incorporation did not preclude the sequestration of soil-C, as is often assumed, but rather assisted in the thorough mixing of soil to improve microbial contact with residue-C and nutrient sources. These observations also suggest a need for reconsideration of crop nutrition approaches in no-till farming systems, where crop residues remain on the soil surface, if increased C-sequestration is an expected outcome.

We gratefully acknowledge an anonymous reviewer whose insightful comments which greatly improved the manuscript.

## Supporting Information

S1 Table Crop biomass and nutrient data. (XLSX)

S2 Table Nutrients added to facilitate humification of 30% of stubble-C. (XLSX)

S3 Table Soil nutrient concentrations (%), loads (t ha-1) and ratios (C:N, C:P, and C:S) in 2006 and 2012. (XLSX)

S4 Table Fertiliser nutrients added for normal crop requirements and NPS mass balance example. (XLSX)
